# Fabrication of Flexible Films for Supercapacitors Using Halloysite Nano-Clay Incorporated Poly(lactic acid)

**DOI:** 10.3390/polym15234587

**Published:** 2023-11-30

**Authors:** Bipin S. Chikkatti, Ashok M. Sajjan, Nagaraj R. Banapurmath, Javed Khan Bhutto, Rajesh Verma, T. M. Yunus Khan

**Affiliations:** 1 Department of Chemistry, KLE Technological University, Hubballi 580031, India; bipinchikkatti@gmail.com; 2 Centre of Excellence in Material Science, School of Mechanical Engineering, KLE Technological University, Hubballi 580031, India; nr_banapurmath@kletech.ac.in; 3 Department of Electrical Engineering, College of Engineering, King Khalid University, Abha 61421, Saudi Arabia; jbhutto@kku.edu.sa (J.K.B.); rkishor@kku.edu.sa (R.V.); 4 Department of Mechanical Engineering, College of Engineering, King Khalid University, Abha 61421, Saudi Arabia

**Keywords:** polymer films, supercapacitor, specific capacitance, energy density, power density

## Abstract

In the past few years, significant research efforts have been directed toward improving the electrochemical capabilities of supercapacitors by advancing electrode materials. The present work signifies the development of poly(lactic acid)/alloysite nano-clay as an electrode material for supercapacitors. Physico-chemical characterizations were analyzed by Fourier transform infrared spectroscopy, wide-angle X-ray diffraction, and a universal testing machine. Cyclic voltammetry, electrochemical impedance spectroscopy, and galvanostatic charge–discharge techniques were employed to evaluate electrochemical characteristics. The optimized poly(lactic acid)/halloysite nano-clay film revealed the highest specific capacitance of 205.5 F g^−1^ at 0.05 A g^−1^ current density and showed 14.6 Wh kg^−1^ energy density at 72 W kg^−1^ power density. Capacitance retention of 98.48% was achieved after 1000 cycles. The microsupercapacitor device presented a specific capacitance of 197.7 mF g^−1^ at a current density of 0.45 mA g^−1^ with 10.8 mWh kg^−1^ energy density at 549 mW kg^−1^ power density.

## 1. Introduction

The majority of the exponentially rising worldwide energy demands is being fulfilled by fossil fuels [1]. Fossil fuel exhaustion and its potentially harmful effects on the ecosystem are forcing the scientific community to create clean and environmentally friendly energy sources [2]. A worldwide goal is to now produce and store energy efficiently while also being environmentally friendly and long-lasting [3]. In contrast to batteries, supercapacitors (SCs) possess the capability to store and release energy at notably elevated rates. This superior performance stems from their energy storage mechanisms, which hinge on a simple process of charge separation occurring at the junction between the electrode and the electrolyte [4]. SCs are highly desirable green energy storage devices with various benefits for enhancing or replacing batteries due to their lightweight, high performance, extended cycle life stability, and high-power density qualities [5]. Although SCs have higher energy densities than ordinary capacitors, they are still far lower than those of batteries or fuel cells [6]. Electrodes, electrolytes, and separators are the three essential parts of SCs. Much effort has gone into raising the specific capacitance of electrodes in SCs to improve performance [7]. Nanostructured materials with greater surface areas and enhanced capacitive performance properties are sought after for SC electrodes. It is currently acknowledged that the creation of nanoengineered materials has noteworthy roles in the development of high-performance SC devices [8].

While having a similar morphology to multiwalled carbon nanotubes, halloysite nano-clay (HNC) is a low-cost, hydrated polymorph made of clay (kaolin group) and phyllosilicate in a 1:1 ratio [9]. The chemical formula of HNC is Al_2_Si_2_O_5_(OH)_4_, and it is a naturally occurring tubular material. The outside surface of HNC is mostly made of SiO_2_, while the inner surface is made of Al_2_O_3_, making the two sides oppositely charged [10]. Inner and outer hydroxyl groups, which are located between layers and on the surface of the nano-clay, respectively, are two different types of hydroxyl groups that exist in HNCs. O-Si-O groups and the siloxane surface make up the majority of the HNC surface [11]. HNC is used in a variety of applications, including wastewater treatment [12], catalysis [13], food packaging [14], drug delivery [15], and wound healing [16]. Lin et al. in 2016 reported that HNC could be used as an electrolyte material in solid-state lithium–sulfur batteries [17]. Zhu et al. in 2018 reported that HNC could be used as an active material in thermal energy storage [18]. Due to its distinctive qualities, such as a tubular nanostructure, high stability, biocompatibility, affordability, and suitable mechanical properties, HNC in polymer composites results in a variety of benefits and may eventually replace extremely expensive CNTs in the production of high-performance composites and multifunctional nanocomposites [19].

Poly(lactic acid) (PLA) is a type of polyester that can be made either chemically or through carbohydrate fermentation using the lactic acid monomer [20]. Due to its high transparency, strong mechanical qualities, moderate water resistance, commercial availability at a reasonable price, biodegradability, and biocompatibility, PLA has applications in food packaging [21], tissue engineering [22], pervaporation [23], textiles [24], and biomedical applications [25]. Peng et al. in 2012 reported that PLA could be used as an electrode material for solar cell applications [26]. Culebras et al. in 2019 reported that PLA had applicability as an electrode material in lithium-ion battery applications [27]. Also, being biodegradable, PLA is recyclable and a compostable material that contributes to a safe environment for human life by being free of greenhouse effects and waste disposal problems. Hence, to create flexible HNC-based composite film electrodes that are degradable and biodegradable, PLA was used as a substrate. HNC also exhibits excellent dispersion in the PLA matrix which is an important behavior when improving PLA/HNC film properties.

In this work, we established flexible PLA–HNC films as electrodes for SC applications. The characterization and electrochemical performance of PLA–HNC films were both performed. Based on excellent electrochemical performances, we fabricated a supercapacitor device and its electrochemical behaviors were evaluated.

## 2. Materials and Methods

### 2.1. Materials

Sigma Aldrich Company, St. Louis, MI, USA, supplied the halloysite nano-clay (with a molecular weight of approximately 294.2), while Nature Works, Blair, NE, USA, provided the poly(lactic acid). Spectrum Reagents and Chemicals, Cochin, India, procured sulfuric acid and chloroform solutions. S.D. Fine Chem Ltd., Mumbai, India, supplied the poly(vinyl alcohol) with a molecular weight of around 124,000.

### 2.2. Preparation of PLA–HNC Films

**Figure 1:**
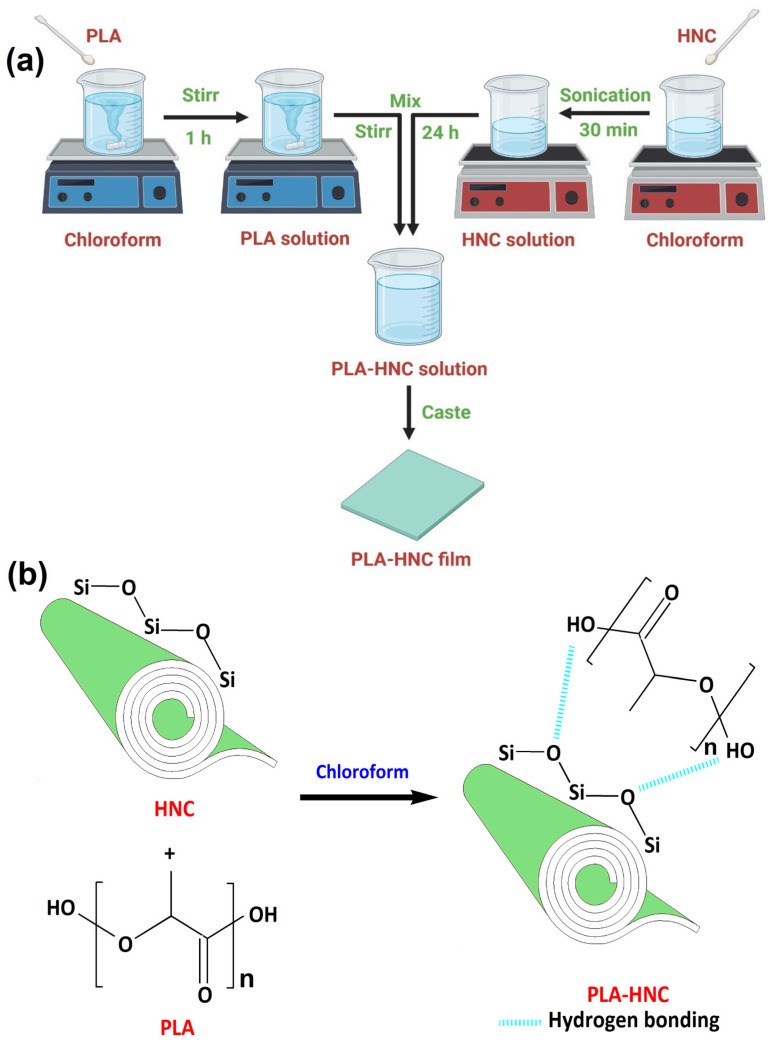
(**a**) Scheme showing the synthesis of PLA–HNC films and (**b**) possible interaction between PLA and HNC.

A solution containing 4 wt% of PLA was prepared by dissolving it in chloroform. The solution was then subjected to continuous stirring for 1 h. The resulting solution was meticulously poured onto a clean glass substrate in an environment devoid of pollutants. The film was allowed to fully dry at ambient temperature. A wholly dried film was peeled off and named F-0. The HNC solution was made by adding a known amount of HNC into chloroform and sonication for 30 min. By introducing the necessary quantity of HNC solution into the PLA solution and agitating for 24 h, a uniform mixture was achieved. To investigate the impact of HNC on PLA, films were fabricated by blending the PLA solution with different HNC percentage (1%, 2%, 3%, and 4%) solutions which were designated as F-1, F-2, F-3, and F-4, respectively. Figure 1a and Figure 1b represent the formulation method for HNC/PLA films and possible interactions between PLA and HNC, respectively.

### 2.3. Fabrication of the SC Device

**Figure 2:**
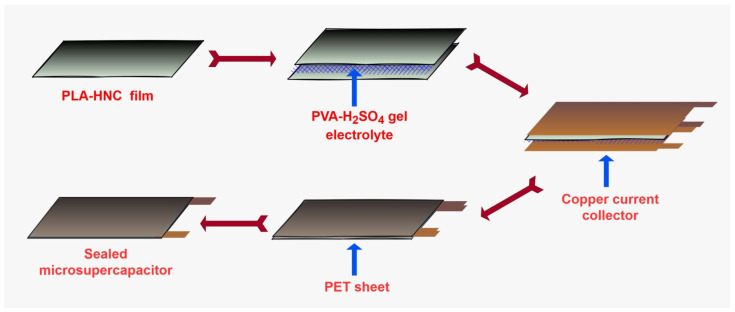
Fabrication method for a sandwich-type microsupercapacitor device using electrode films, a gel electrolyte, and a copper current collector.

Sandwich-type supercapacitors are a type of supercapacitor that consist of two or more electrodes that are separated by a thin layer of liquid electrolyte or gel that contains ions. Sandwich-type supercapacitors are known for their high power density and fast charging times. Figure 2 shows the fabrication of a sandwich-type microsupercapacitor device. Developed PLA–HNC films were cut into rectangular shapes of 5 cm × 7.5 cm. Two such films were sandwiched between PVA-H_2_SO_4_ gel electrolytes and left overnight to dry. Resultant films were sandwiched between copper metal foil that provided an electrical connection between the SC device and the electrochemical workstation. A polyethylene terephthalate (PET) sheet device was sealed by heat action. To create the PVA-H_2_SO_4_ gel electrolyte, 5 g of PVA granules were combined with 50 mL of a 10 wt% aqueous solution of H_2_SO_4_. To obtain a clear, homogeneous solution, it was heated at 70 °C for a 2 h period. The solution was then gradually cooled and used while fabricating the SC device. The PVA-H_2_SO_4_ gel electrolyte provides a path for the movement of ions between the electrodes, which is essential for the charge–discharge process. It also provides mechanical support to electrodes, prevents them from short-circuiting, and prevents the leakage of liquid electrolytes, which can damage the supercapacitor and shorten its lifespan.

### 2.4. Electrochemical Characterizations of PLA–HNC Films

Using a CHI660E electrochemical workstation from CH Instruments, Bee Cave, TX, USA, cyclic voltammetry (CV), electrochemical impedance spectroscopy (EIS), and galvanostatic charge-discharge (GCD) analyses were conducted on PLA–HNC films employed as electrodes. These tests were performed in an H_2_SO_4_ (1 M) electrolyte, utilizing a standard two-electrode configuration. Different scan rates (5–100 mV s^−1^) were used to evaluate CV in the potential range from −0.8 to +0.8 V. A 5 mV amplitude and a frequency range of 1–100 kHz were used to analyze EIS measurements. Variable current densities (0.05–0.125 A g^−1^) were used to run GCD. Using cyclic voltammograms, the specific capacitance of films was calculated using Equation (1). Using charge–discharge curves, the specific capacitance of films was calculated using Equation (2). Also, the energy density and power density of films with the help of GCD curves were calculated utilizing Equations (3) and (4), respectively.

C = 2∫IdV/m∆VS(1)

C = 4I∆t/m∆V(2)

E = C(∆V)^2^/2 × 3600 (3)

P = 3600E/∆t(4)

In this context, C represents the specific capacitance, ∫IdV corresponds to the enclosed region beneath the curve, m denotes the mass of the electrodes, ∆V stands for the potential range, S signifies the scan rate, I indicates the current observed during discharge, ∆t represents the duration of discharge, E symbolizes the energy density, and P represents the power density.

## 3. Results

### 3.1. The Physico-Chemical Characterization of PLA–HNC Films

**Figure 3:**
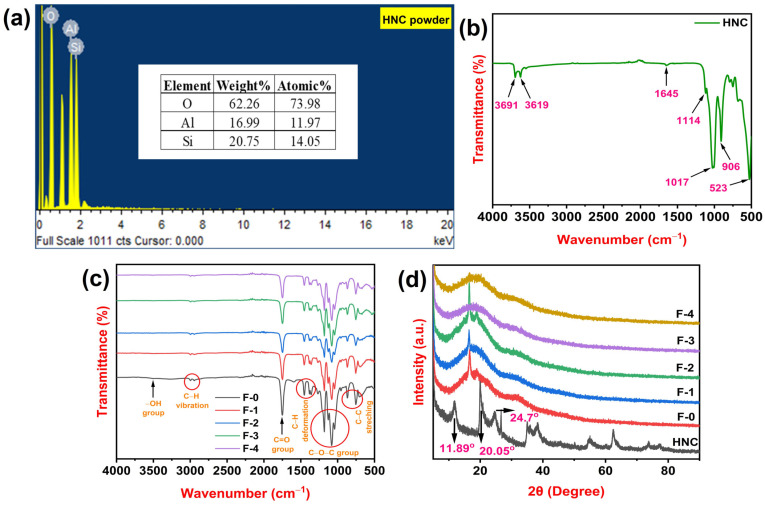
(**a**) EDS of HNC powder (**b**) FTIR spectrum of plane HNC (**c**) FTIR spectra of 1 wt% HNC/PLA (F-1), 2 wt% HNC/PLA (F-2), 3 wt% HNC/PLA (F-3), and 4 wt% HNC/PLA (F-4) (**d**) WAXD patterns of plane HNC, 1 wt% HNC/PLA (F-1), 2 wt% HNC/PLA (F-2), 3 wt% HNC/PLA (F-3), and 4 wt% HNC/PLA (F-4).

Energy-dispersive X-ray spectroscopy (EDS) is a microanalytical technique used to identify and quantify the elemental composition of a sample. Figure 3a illustrates the EDS (Zeiss Ultra 55 Gemini Oxford Instruments, X-Max, Oberkochen, Germany) results of the HNC powder and shows that halloysite nano-clay is predominantly composed of oxygen, silicon, and aluminum with weights% of 62.26, 16.99 and 14.05, respectively [28].

Fourier transform infrared spectroscopy (FTIR) is a powerful analytical technique that uses infrared light to identify and quantify molecules. Figure 3b represents the FTIR (FTIR Spectrometer, PerkinElmer spectrum, Singapore) spectrum of plane HNC. Internal and exterior HNC hydroxyl groups are responsible for peaks at 3691 cm^−1^ and 3619 cm^−1^. The deformation vibration at 1645 cm^−1^ indicates interlayer water [29]. The peak at 1114 cm^−1^ is signified by the apical Si−O’s stretching mode, whereas the band at 1017 cm^−1^ is brought on by the stretching vibrations of Si−O−Si. Al−O bending mode is represented by the band at 906 cm^−1^, and the peak at 523 cm^−1^ is caused by the vibration of Al−O−Si [30]. Figure 3c displays the FTIR spectra of PLA and HNC-incorporated PLA films. The −OH group’s distinctive band is seen at 3516 cm^−1^. The CH_3_ group’s symmetric and asymmetric C-H vibrations are attributed to bands at 2999 and 2939 cm^−1^, respectively [31]. An important characteristic of ester bonds is C=O stretching at 1748 cm^−1^ for PLA. Multiple bands at 1454, 1381, and 1361 cm^−1^ are related to C-H deformations. C–O–C stretching causes peaks at 1185, 1128, 1080, and 1041 cm^−1^. Small peaks at 869 and 755 cm^−1^ are signified by C–O–C stretching vibrations [32]. As the HNC content increases, a marginal decrease in the intensity of the O-H stretching band of PLA is noted. This is due to the hydrogen bonding of the O-H group of PLA to the Si-O group of HNC [33]. A minor decrease in intensity is noted because the –OH groups of PLA also interact among themselves, forming hydrogen bonds.

Wide-angle X-ray diffraction (WAXD) is a non-destructive analytical technique that uses X-rays to determine the crystal structure, phase composition, and crystallinity of materials. Using WAXD (Philips Analytical Ultima IV X-ray diffraction system, SmartLab SE Rigaku, Tokyo, Japan) investigations, the impact of HNC loading on PLA films was investigated. Figure 3d represents diffractograms of HNC, plane PLA (F-0), 1 wt% HNC/PLA (F-1), 2 wt% HNC/PLA (F-2), 3 wt% HNC/PLA (F-3), and 4 wt% HNC/PLA (F-4). The characteristic diffraction peaks at 2θ = 24.7°, 20.05°, and 11.89° can be attributed to the crystalline nature of HNC [34,35]. In contrast to amorphous polymers, which have wide diffraction peaks, crystalline polymers have crisp peaks with high intensities. From WAXD patterns observed for plane PLA (F-0), we found a peak at 2θ = 16.5° that corresponded to the degree of crystallinity of PLA [36]. Diffraction peaks at 2θ = 16.6°, 16.7°, 17.6°, and 18.4° correspond to 1 wt% HNC/PLA (F-1), 2 wt% HNC/PLA (F-2), 3 wt% HNC/PLA (F-3), and 4 wt% HNC/PLA (F-4), respectively. As the HNC content increased in the PLA matrix, the intensity of the diffraction peaks gradually decreases and peaks are shifted to higher 2θ angles. This is because of HNC assimilation into the PLA substrate, thereby increasing interactions between HNC and the PLA matrix.

The mechanical strength and elastic nature of developed films were both analyzed using the Universal Testing Machine (UTM) (DAK system Inc., Maharastra, India). Table 1 provides information on tensile strength, Young’s modulus, and elongation break values in fabricated films. Plane PLA is very rigid and brittle. The tensile strength is 24 MPa and Young’s modulus is 1988 MPa, whereas elongation at break is 2.9%. As the HNC content in PLA increases from 1 wt% HNC/PLA (F-1) to 2 wt% HNC/PLA (F-2), tensile strength and Young’s modulus increase, whereas elongation break decreases. This is attributed to large numbers of hydrogen bonds between PLA and HNC. Large hydrogen bond formations result in robust films with high tensile strength and Young’s modulus because hydrogen bonds increase film resistance to external forces. With a high Young’s modulus, the PLA chains’ resistance to elastic deformation is increased by strong hydrogen bonds that limit their mobility. As the HNC concentration in PLA increases from 3 wt% HNC/PLA (F-3) to 4 wt% HNC/PLA (F-4), tensile strength and Young’s modulus decrease, whereas elongation break values increase due to HNC agglomeration in PLA films. Agglomeration decreases the amount of HNC–PLA contact surface areas, which in turn decrease the quantity of hydrogen bonds between PLA and HNC. Additionally, HNC agglomeration causes free volumes to accumulate inside the PLA, increasing the amount of space available for PLA chain mobility as well as for elastic and plastic deformation. Agglomeration also leads to the production of weak spots with low tensile strength and Young’s modulus, which are easily shattered by an outside force [37]. These results demonstrate that HNC concentrations added to the PLA matrix have an impact on the mechanical characteristics of HNC/PLA nanocomposite films because they have an impact on HNC dispersion inside the PLA matrix.

### 3.2. Electrochemical Performances of PLA–HNC Films

**Figure 4:**
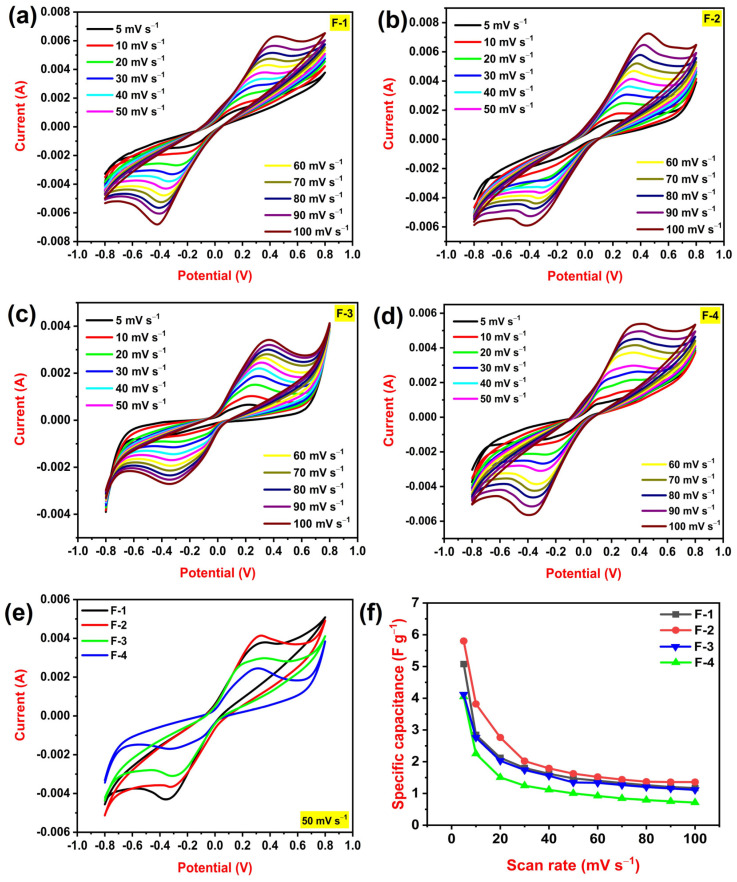
(**a**–**d**) CV curves at variable scan rates (**e**) CV curves at a fixed scan rate of 50 mV s^−1^ (**f**) variation in specific capacitance concerning the scan rate of 1 wt% HNC/PLA (F-1), 2 wt% HNC/PLA (F-2), 3 wt% HNC/PLA (F-3), and 4 wt% HNC/PLA (F-4).

The electrochemical performance of supercapacitors is important because it determines their ability to store and deliver energy. CV is an electrochemical technique used to study the redox properties of a chemical species. It involves applying a linearly changing potential to an electrochemical cell and measuring the resulting current. One of the more well-known dynamic electrochemical techniques CV scans the potential provided to an electrochemical cell [38]. Figure 4a–d illustrates the cyclic voltammograms of all PLA–HNC films at different scan rates from 5 mV s^−1^ to 100 mV s^−1^. From graphs, it is noted that as scan rates increase the area under the curve also increases [39]. The area under the curve is the least for 5 mV s^−1^ and the maximum for 100 mV s^−1^. All CV curves show visible redox peaks which suggest that the developed films show a pseudocapacitive nature. It is a faradaic process, meaning that the process involves the transfer of electrons between the electrode and electrolyte ions. It is characterized by a fast and reversible redox reaction. The possible redox reaction of halloysite nano-clay is as follows:

Al_2_Si_2_O_5_(OH)_4_ + H^+^ + e^−^ ↔ [Al_2_Si_2_O_5_(OH)_3_]^−^ + H_2_O (5)

In this reaction, aluminum ions in nano-clay are reduced from a +3 to a +2 oxidation state. The redox reaction of halloysite nano-clay can be used to store energy in the form of electrons. This is because electrons can be released from the halloysite when it is oxidized. Figure 4e displays CV curves for all PLA–HNC films at a fixed scan rate of 50 mV s^−1^. It is noted that the area beneath the curve is maximum for 2 wt% HNC/PLA (F-2) with an anodic peak current of 0.00414 A. Later, we calculate the specific capacitance of all developed PLA–HNC films at different scan rates using Equation (1). Figure 4f displays the variations in the specific capacitance of all films with respect to scan rates. At all scan rates, the specific capacitance of 2 wt% HNC/PLA (F-2) is maximum. The 2 wt% HNC/PLA (F-2) recorded the highest specific capacitance of 5.8 F g^−1^ at 5 mV s^−1^ and a minimum specific capacitance of 1.3 F g^−1^ at 100 mV s^−1^. As the scan rate increases, the specific capacitance of the material decreases. The fundamental factor for the decline in specific capacitance with increasing scan rate is the electrode material’s insufficient ability to access electrons. From the CV technique, we arrived at the conclusion that 2 wt% HNC/PLA (F-2) is the optimized film due to its elevated electrochemical performance.

**Figure 5:**
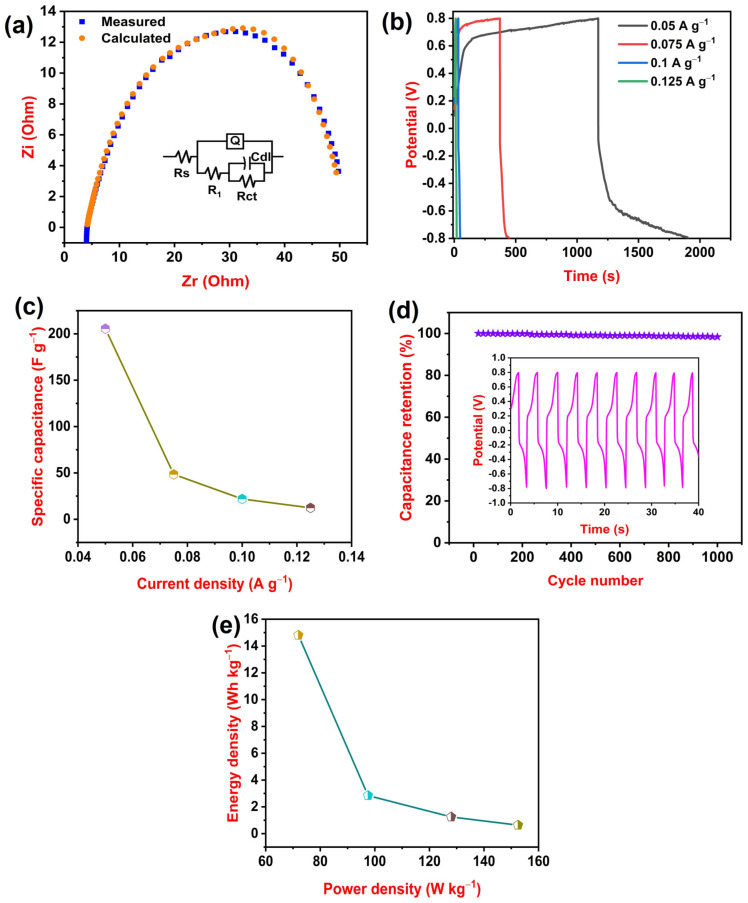
(**a**) Nyquist plot along with a model of an equivalent circuit (**b**) charge–discharge curves at variable current densities (**c**) variation in specific capacitance concerning current density (**d**) cycle life up to 1000 cycles and (**e**) a Ragon plot.

EIS is a powerful electrochemical technique that can be used to study the properties of materials and electrode reactions. EIS involves applying a small amplitude AC potential to an electrochemical system and measuring the resulting AC current. EIS is a useful experimental method for determining a system’s frequency response [40]. Figure 5a illustrates the Nyquist plot for the 2 wt% HNC/PLA (F-2) film. In the plot, the real part of the impedance is plotted on the x-axis and the imaginary part of the impedance is plotted on the y-axis [41]. For fitting, we used ZsimpWin 3.21 software and analyzed different impedance parameters using equivalent circuit model Rs(Q(R_1_(CdlRct))), where R, Q, and Cdl represent resistance, constant phase element, and electrical double-layer capacitance, respectively.

Solution resistance refers to the opposition to current flow through an electrolyte solution by ion migration. Charge transfer resistance is a measure of the resistance to the transfer of electrons across an interface, such as between an electrode and an electrolyte in an electrochemical cell. A constant phase element is a circuit element that has a frequency-independent phase angle between current and voltage. Electrical double-layer capacitance is the ability of a surface to store electrical energy by creating an electrical double layer at its interface with an electrolyte. The electrical double layer is a region where two layers of oppositely charged ions form, one on the surface of the electrode and one in the electrolyte. The two layers are separated by a thin layer of solvent molecules. In particular, lower solution resistance (Rs) and charge transfer resistance (Rct) findings enable a good transfer of electrons between electrode and electrolyte [42]. Rs, Q, R_1_, Cdl, and Rct findings were 4.101 Ohm, 0.0004542 S-s^n^ (n = 0.6929), 37.42 Ohm, 0.001053 F, and 9.783 Ohm, respectively.

Galvanostatic tests stand out among electrochemical characterization techniques typically employed in research involving films because they offer direct access to the contribution of a cell’s overall voltage in various film states [43]. Figure 5b illustrates GCD curves for 2 wt% HNC/PLA (F-2) at 0.05, 0.075, 0.1, and 0.125 A g^−1^ current density.

As the current density decreases, the time taken for charge–discharge increases. This is because the current density is the rate of charge flow per unit area, and so a lower current density means that it will take longer to charge or discharge the same amount of charge. At 0.05 A g^−1^ current density, the time taken to charge–discharge was 1906 s, whereas at 0.125 A g^−1^, the current density time taken was 16 s. Furthermore, we calculated the specific capacitance of 2 wt% HNC/PLA (F-2) at different current densities using Equation (2). Figure 5c displays the variation in specific capacitance at different current densities. From the graph, it is noted that 2 wt% HNC/PLA (F-2) achieved a maximum specific capacitance of 205.5 F g^−1^ at 0.05 A g^−1^ and a minimum specific capacitance of 12.3 F g^−1^ at 0.125 A g^−1^ current density. Huige et al. in 2022 reported that a PANI-PLA electrode material showed areal capacitance of 5 mF cm^−2^ at 0.1 mA cm^−2^ [44], Ramadass et al. in 2020 reported that an AHNC electrode material in aqueous electrolyte exhibited specific capacitance of 197 F g^−1^ at 0.3 A g^−1^ [45], and Jekal et al. in 2023 reported that carbonized MnO_2_/PLA and Fe_3_O_4_/PLA displayed an areal capacitance of 34.8 mF cm^−2^ and 47.9 mF cm^−2^ at 1 mA cm^−2^, respectively [46], whereas our PLA–HNC electrode material displayed a significant specific capacitance of 205.5 F g^−1^ at 0.05 A g^−1^. Cycle study is the process of evaluating the performance of a supercapacitor over repeated charge and discharge cycles. It is an important part of supercapacitor development and research as it can provide valuable information about capacity retention. Figure 5d displays a cycle study of 2 wt% HNC/PLA (F-2) at current density 0.125 A g^−1^ with a potential vs. time graph for the first 10 cycles. After prolonged 1000 cycles, 98.48% capacitance retention was found. Later, we calculate the energy density and power density of 2 wt% HNC/PLA (F-2) using Equations (3) and (4), respectively. A Ragon plot is a graph that compares the energy density and power density of energy storage devices. Figure 5e represents a Ragon plot where the variation in energy density is measured with power density. From the graph, it is noted that an energy density of 14.6 Wh kg^−1^ is achieved at a power density of 72 W kg^−1^ and energy density decreased to 0.63 Wh kg^−1^ when the power density increased to 152.5 W kg^−1^. These results underscore the prospective utility of our PLA/HNC film as a promising electrode material for supercapacitors.

**Figure 6:**
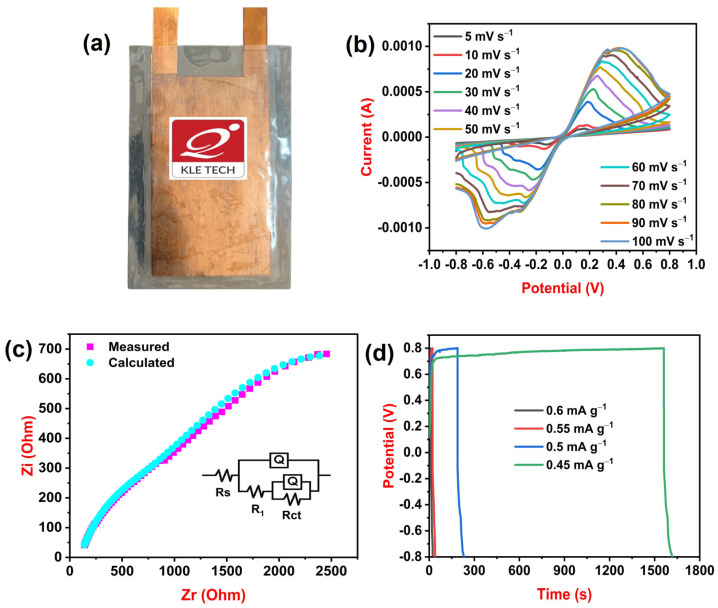
(**a**) Photo of a microsupercapacitor device (**b**) cyclic voltammogram of an SC device at different scan rates (**c**) impedance curves with an equivalent circuit of SC device and (**d**) charge–discharge curves of the SC device at different current densities.

CV, EIS, and GCD tests are used to analyze the electrochemical behavior of fabricated SC devices. A photo image of the SC device is provided in Figure 6a and contains 2 wt% HNC/PLA (F-2) as electrodes, PVA-H_2_SO_4_ gel as an electrolyte, and copper foil as a current collector. Copper current collectors collect and distribute current between the active electrode material and the external circuit. This is essential for efficient charge and discharge of the supercapacitor. Figure 6b illustrates the CV data recorded at different scan rates from 5 to 100 mV s^−1^. Redox peaks are observed in the graph, which suggest pseudocapacitance behavior. A specific capacitance of 37.3 mF g^−1^ is recorded at a scan rate of 5 mV s^−1^. A Nyquist plot with an equivalent circuit model is displayed in Figure 6c to measure the impedance behavior of the device. Equivalent circuit model Rs(Q(R_1_(Q(RctW)))) is used to fit impedance data, where R, Q, and W represent resistance, constant phase element, and Warburg impedance. Impedance parameters of the device are Rs-93.39 Ohm, Q-1.848 × 10^−5^ S-s^n^ (n = 0.5), R_1_-922.9 Ohm, Q-0.000139 S-s^n^ (n = 0.4), Rct-3387 Ohm, and W-3.063 × 10^6^ S-s^5^. Figure 6d displays GCD curves of the SC device at different current densities from 0.6 to 0.45 mA g^−1^. The SC device displays exceptional specific capacitance of 197.7 mF g^−1^ at 0.45 mA g^−1^ current density and decreases to 23.3 mF g^−1^ at 0.6 mA g^−1^ current density. We also calculated the energy density and power density of the SC device. An energy density of 1.6 mWh kg^−1^ is attained at 864 mW kg^−1^ power density and energy density increases to 10.8 mWh kg^−1^ at 549 mW kg^−1^ power density. When a microsupercapacitor device is bent to 60°, we observe a slight decrease in specific capacitance of about 2% because bending can disrupt ion transport pathways in the electrolyte, making it more difficult for ions to reach electrode surfaces. All these electrochemical findings suggest that HNC-incorporated PLA films are promising electrodes for supercapacitors.

## 4. Conclusions

In summary, we developed HNC-incorporated PLA films as electrode materials for supercapacitor applications. The hydrogen bonding between PLA and HNC results in a decrease in the peak intensity of the −OH group in FTIR spectra. The presence of HNC in the PLA matrix is also confirmed by the WAXD technique whereupon HNC addition, the intensity of the diffraction peaks gradually decreased and peaks were shifted to higher 2θ angles. UTM suggests that HNC can alter the mechanical properties of PLA films. Among our developed films, 2 wt% HNC/PLA (F-2) is considered an optimized film as an electrode due to the maximum area under the curve in cyclic voltammograms with the highest anodic peak current (0.00414 A) and specific capacitance of 5.8 F g^−1^ at 5 mV s^−1^ scan rate. Using the EIS technique, lower Rs (4.101 Ohm), Rct (9.783 Ohm), and higher Cdl (0.001053 F) values were found. Using GCD technique parameters like specific capacitance, a cycle study, energy density, and power density were recorded. Our 2 wt% HNC/PLA (F-2) displayed a maximum specific capacitance of 205.5 F g^−1^ at 0.05 A g^−1^ current density. An energy density of 14.6 Wh kg^−1^ was achieved at a power density of 72 W kg^−1^. After 1000 cycles, 98.48% capacitance retention was found. By analyzing the electrochemical behavior of the 2 wt% HNC/PLA (F-2) film, a better SC device was fabricated and tested. The SC device displayed a specific capacitance of 197 mF g^−1^ at 0.45 mA g^−1^ with an exceptional energy density of 10.8 mWh kg^−1^ at a power density of 549 mW kg^−1^, thereby generating a promising electrode material for supercapacitor applications.

## Figures and Tables

**Table 1:** Mechanical properties of HNC-incorporated PLA films.

Films	Tensile Strength (MPa)	Young’s Modulus (MPa)	Elongation at Break (%)
F-0	24 ± 1.2	1988 ± 3.2	2.9 ± 0.5
F-1	27 ± 2.3	4072 ± 2.1	2.2 ± 0.7
F-2	30 ± 1.9	8267 ± 3.4	1.5 ± 1.1
F-3	28 ± 2.3	7071 ± 3.7	1.8 ± 1.2
F-4	25 ± 2.4	5373 ± 1.9	2.5 ± 0.9

## Data Availability

The data presented in this study are available on request from the corresponding author.

## References

[1] Larcher D., Tarascon J.-M. (2015). Towards Greener and More Sustainable Batteries for Electrical Energy Storage. Nat. Chem..

[2] Ordowich C., Chase J., Steele D., Malhotra R., Harada M., Makino K. (2012). Applying Learning Curves to Modeling Future Coal and Gas Power Generation Technologies. Energy Fuels.

[3] Zhang Y.-Z., Wang Y., Cheng T., Lai W.-Y., Pang H., Huang W. (2015). Flexible Supercapacitors Based on Paper Substrates: A New Paradigm for Low-Cost Energy Storage. Chem. Soc. Rev..

[4] Iro Z.S., Subramani C., Dash S.S. (2016). A Brief Review on Electrode Materials for Supercapacitor. Int. J. Electrochem. Sci..

[5] Laelabadi K.G., Moradian R., Manouchehri I. (2021). Facile Method of Fabricating Interdigitated and Sandwich Electrodes for High-Performance and Flexible Reduced Graphene Oxide@Polyaniline Nanocomposite Supercapacitors. ACS Appl. Energy Mater..

[6] Zhang L.L., Zhao X.S. (2009). Carbon-Based Materials as Supercapacitor Electrodes. Chem. Soc. Rev..

[7] Vargun E., Ozaltin K., Fei H., Harea E., Vilčáková J., Kazantseva N., Saha P. (2020). Biodegradable Porous Polylactic Acid Film as a Separator for Supercapacitors. J. Appl. Polym. Sci..

[8] Vangari M., Pryor T., Jiang L. (2013). Supercapacitors: Review of Materials and Fabrication Methods. J. Energy Eng..

[9] Idumah C.I., Hassan A., Ogbu J., Ndem J., Nwuzor I.C. (2019). Recently Emerging Advancements in Halloysite Nanotubes Polymer Nanocomposites. Compos. Interfaces.

[10] Yendluri R., Otto D.P., De Villiers M.M., Vinokurov V., Lvov Y.M. (2017). Application of Halloysite Clay Nanotubes as a Pharmaceutical Excipient. Int. J. Pharm..

[11] Kamble R., Ghag M., Gaikawad S., Kumar Panda B. (2012). Halloysite Nanotubes and Applications: A Review. J. Adv. Sci. Res..

[12] Ağtaş M., Dilaver M., Koyuncu I. (2021). Halloysite Nanoclay Doped Ceramic Membrane Fabrication and Evaluation of Textile Wastewater Treatment Performance. Process. Saf. Environ. Prot..

[13] Sadjadi S. (2020). Halloysite-Based Hybrids/Composites in Catalysis. Appl. Clay Sci..

[14] Bumbudsanpharoke N., Ko S. (2019). Nanoclays in Food and Beverage Packaging. J. Nanomater..

[15] Lvov Y.M., DeVilliers M.M., Fakhrullin R.F. (2016). The Application of Halloysite Tubule Nanoclay in Drug Delivery. Expert Opin. Drug Deliv..

[16] Pavliňáková V., Fohlerová Z., Pavliňák D., Khunová V., Vojtová L. (2018). Effect of Halloysite Nanotube Structure on Physical, Chemical, Structural and Biological Properties of Elastic Polycaprolactone/Gelatin Nanofibers for Wound Healing Applications. Mater. Sci. Eng. C.

[17] Lin Y., Wang X., Liu J., Miller J.D. (2017). Natural Halloysite Nano-Clay Electrolyte for Advanced All-Solid-State Lithium-Sulfur Batteries. Nano Energy.

[18] Zhu X., Shchukin D. (2018). Crystallohydrate Loaded Halloysite Nanocontainers for Thermal Energy Storage. Adv. Eng. Mater..

[19] Ma W., Wu H., Higaki Y., Takahara A. (2018). Halloysite Nanotubes: Green Nanomaterial for Functional Organic-Inorganic Nanohybrids. Chem. Rec..

[20] Tawakkal I.S.M.A., Cran M.J., Miltz J., Bigger S.W. (2014). A Review of Poly(Lactic Acid)-Based Materials for Antimicrobial Packaging. J. Food Sci..

[21] Roy S., Rhim J.-W. (2020). Preparation of Bioactive Functional Poly(Lactic Acid)/Curcumin Composite Film for Food Packaging Application. Int. J. Biol. Macromol..

[22] Armentano I., Bitinis N., Fortunati E., Mattioli S., Rescignano N., Verdejo R., Lopez-Manchado M., Kenny J. (2013). Multifunctional Nanostructured PLA Materials for Packaging and Tissue Engineering. Prog. Polym. Sci..

[23] Rhim J., Lee S., Kim Y. (2002). Pervaporation Separation of Water–Ethanol Mixtures Using Metal-Ion-Exchanged Poly(Vinyl Alcohol) (PVA)/Sulfosuccinic Acid (SSA) Membranes. J. Appl. Polym. Sci..

[24] Yang Y., Zhang M., Ju Z., Tam P.Y., Hua T., Younas M.W., Kamrul H., Hu H. (2021). Poly(Lactic Acid) Fibers, Yarns and Fabrics: Manufacturing, Properties and Applications. Text. Res. J..

[25] Singhvi M.S., Zinjarde S.S., Gokhale D.V. (2019). Polylactic Acid: Synthesis and Biomedical Applications. J. Appl. Microbiol..

[26] Peng S., Zhu P., Wu Y., Mhaisalkar S.G., Ramakrishna S. (2012). Electrospun Conductive Polyaniline–Polylactic Acid Composite Nanofibers as Counter Electrodes for Rigid and Flexible Dye-Sensitized Solar Cells. RSC Adv..

[27] Culebras M., Geaney H., Beaucamp A., Upadhyaya P., Dalton E., Ryan K.M., Collins M.N. (2019). Bio-derived Carbon Nanofibres from Lignin as High-Performance Li-Ion Anode Materials. ChemSusChem.

[28] Bediako E.G., Nyankson E., Dodoo-Arhin D., Agyei-Tuffour B., Łukowiec D., Tomiczek B., Yaya A., Efavi J.K. (2018). Modified Halloysite Nanoclay as a Vehicle for Sustained Drug Delivery. Heliyon.

[29] Zhang W., Mu B., Wang A. (2015). Halloysite Nanotubes Template-Induced Fabrication of Carbon/Manganese Dioxide Hybrid Nanotubes for Supercapacitors. Ionics.

[30] Barot T., Rawtani D., Kulkarni P. (2020). Physicochemical and Biological Assessment of Silver Nanoparticles Immobilized Halloysite Nanotubes-Based Resin Composite for Dental Applications. Heliyon.

[31] Chikkatti B.S., Sajjan A.M., Kalahal P.B., Banapurmath N.R., Ayachit N.H. (2023). Fabrication and assessment of poly(lactic acid)-poly(4-styrene sulfonate) flexible membranes as electrodes for supercapacitors. J. Energy Storage.

[32] Tham W.L., Poh B.T., Ishak Z.A.M., Chow W.S. (2015). Water Absorption Kinetics and Hygrothermal Aging of Poly(Lactic Acid) Containing Halloysite Nanoclay and Maleated Rubber. J. Polym. Environ..

[33] Lim K., Chow W.S., Pung S.Y. (2019). Accelerated Weathering and UV Protection-Ability of Poly(Lactic Acid) Nanocomposites Containing Zinc Oxide Treated Halloysite Nanotube. J. Polym. Environ..

[34] Govindaraj D., Rajan M. (2021). Investigation of Minerals Substituted Hydroxyapatite Based Nanocomposite Coated Titanium Implant for Bone Tissue Engineering Applications. Orient. J. Chem..

[35] Dong Y., Haroosh H.J. Processing-Structure-Property Relationship for Electrospun Poly Lactic Acid (PLA)/Halloysite Nanotube (HNT) Composite Mats. Proceedings of the 9th Asian-Australasian Conference on Composite Materials (ACCM-9).

[36] Silverajah V.S.G., Ibrahim N.A., Yunus W.M.Z.W., Hassan H.A., Woei C.B. (2012). A Comparative Study on the Mechanical, Thermal and Morphological Characterization of Poly(Lactic Acid)/Epoxidized Palm Oil Blend. Int. J. Mol. Sci..

[37] Risyon N.P., Othman S.H., Basha R.K., Talib R.A. (2020). Characterization of Polylactic Acid/Halloysite Nanotubes Bionanocomposite Films for Food Packaging. Food Packag. Shelf Life.

[38] Rusling J.F., Suib S.L. (1994). Characterizing Materials with Cyclic Voltammetry. Adv. Mater..

[39] Chikkatti B.S., Sajjan A.M., Kalahal P.B., Banapurmath N.R. (2023). Insight into the Performance of Valve-Regulated Lead-Acid Battery Using Sodium Salt of Poly(4-Styrene Sulfonic Acid-Co-Maleic Acid)-Poly(Vinyl Alcohol) Gel Electrolyte. J. Energy Storage.

[40] Taberna P., Portet C., Simon P. (2006). Electrode Surface Treatment and Electrochemical Impedance Spectroscopy Study on Carbon/Carbon Supercapacitors. Appl. Phys. A Mater. Sci. Process..

[41] Chikkatti B.S., Sajjan A.M., Banapurmath N.R., Ayachit N.H. (2023). Graphene-Doped Hydrogels Promoting Ionic Conductivity in Gel-Valve-Regulated Lead Acid Batteries. Langmuir.

[42] Chikkatti B.S., Sajjan A.M., Kalahal P.B., Banapurmath N.R., Angadi A.R. (2023). Insight into the Performance of VRLA Battery Using PVA-TEOS Hybrid Gel Electrolytes with Titania Nanoparticles. J. Energy Storage.

[43] Barros K.S., Martí-Calatayud M.C., Scarazzato T., Bernardes A.M., Espinosa D.C.R., Pérez-Herranz V. (2021). Investigation of Ion-Exchange Membranes by Means of Chronopotentiometry: A Comprehensive Review on This Highly Informative and Multipurpose Technique. Adv. Colloid Interface Sci..

[44] Wei H., Li G., Wan T., Chen A., Peng Z., Zhang H. (2022). Polyaniline Growing on Polylactic Acid Substrate towards Flexible and Biodegradable Supercapacitors. Fuhe Cailiao Xuebao/Acta Mater. Compos. Sin..

[45] Ramadass K., Sathish C.I., MariaRuban S., Kothandam G., Joseph S., Singh G., Kim S., Cha W., Karakoti A.S., Belperio T. (2020). Carbon Nanoflakes and Nanotubes from Halloysite Nanoclays and Their Superior Performance in CO_2_ Capture and Energy Storage. ACS Appl. Mater. Interfaces.

[46] Jekal S., Kim M.-S., Kim D.-H., Noh J., Kim H.-Y., Kim J., Yi H., Oh W.-C., Yoon C.-M. (2023). Fabrication of Flexible All-Solid-State Asymmetric Supercapacitor Device via Full Recycling of Heated Tobacco Waste Assisted by PLA Gelation Template Method. Gels.

